# The Impact of Patient-Centered, Structured Interdisciplinary Bedside Rounds on Medical Staff Satisfaction, Education, and Experience

**DOI:** 10.7759/cureus.76412

**Published:** 2024-12-26

**Authors:** Amer Aldamouk

**Affiliations:** 1 Internal Medicine, Luminis Health Anne Arundel Medical Center, Annapolis, USA

**Keywords:** hospital based medicine, interdisciplinary rounding, interprofessional education and collaboration, medical staff education, medical staff satisfaction

## Abstract

Background

Daily interdisciplinary rounds in hospitals are becoming standardized to maximize the multidisciplinary approach to hospitalized patient care. We hypothesize that structured Interdisciplinary Bedside Rounds (IDRs) increase the satisfaction, education, and experience of medical staff and thus detail actionable recommendations for IDR implementation or delineate measurable long-term impacts.

Methods

This observational study was performed in a 300-bed community hospital. Participants included internal medicine physicians, nurses, case managers, social workers, physical therapists, clinical pharmacists, and nurse leaders. Surveys were used to assess the impact of structured IDRs on medical education, clinical skills, and experience.

Results

We sent 100 surveys and received 72 completed surveys. Results varied among healthcare providers (physicians, nurses, pharmacists, and case managers), reflecting that physicians and nurses reported the highest benefits. There was a positive response on the impact of multidisciplinary rounds on medical staff education, skills, and experience.

Conclusion

Structured IDRs positively impact healthcare providers' (physicians, nurses, pharmacists, and case managers) medical education, clinical skills, and experience, as reported by participants' responses. Implementing IDRs in the health care system and medical school curriculum may impact medical education, clinical skills, experience, and patient care. However, more studies are required to examine the long-term impact of IDRs

## Introduction

Interprofessional communication (IPC) between healthcare teams is one of the essential components of high-quality patient care [1]. Effective interprofessional communication is linked to improved patient safety, as it helps prevent errors and coordinate care across disciplines [2,3]. One strategy to enhance IPC is Interdisciplinary Bedside Rounds (IDRs) [1].

Hospital medicine is complex and fast-paced, requiring high levels of collaboration among diverse care providers, including physicians, nurses, pharmacists, case managers, and patients [4,5]. To address this need, structured IDRs have emerged as a standardized method to promote high-quality interprofessional care through effective communication [1]. Many healthcare institutions have adopted IDRs to foster teamwork, improve care coordination, enhance communication, and actively involve patients in their care planning [6,7].

The bedside model for IDRs offers a structured process that engages all team members in delivering patient-centered care. IDRs can take place at the patient’s bedside or just outside the room, focusing on real-time feedback and patient involvement in care planning. This approach promotes better alignment of treatment goals and allows for immediate action on issues such as unnecessary interventions (e.g., unnecessary intravenous lines) [8,9]. Collaboration during these rounds helps integrate diverse expertise, improving medical decision-making and advancing care quality for hospitalized patients [10,11].

The literature shows that IDRs are associated with higher levels of patient satisfaction, better teamwork, improved clinical outcomes, and enhanced medical education for healthcare providers [12]. However, despite the evidence supporting their benefits, IDRs are still not widely implemented [13]. Moreover, while several studies have assessed the impact of IDRs on patient outcomes, few have explored how they influence the education, skills, and experiences of healthcare providers.

The present study aims to evaluate providers’ satisfaction, education, experience, and skill levels following their participation in IDRs by using survey-based assessments.

## Materials and methods

Study design

This study is an observational study conducted in a 300-bed community hospital in Mechanicsville, Virginia, USA.

Participants included internal medicine physicians, nursing staff, case managers, social workers, physical therapy representatives, clinical pharmacists, and nurse leaders who supervise interdisciplinary rounds.

The hospital features several specialized care units to address varying levels of patient acuity. The Intermediate Care Unit (IMCU) provides close monitoring for patients requiring advanced interventions, such as continuous intravenous medication administration or respiratory therapy, and maintains a nurse-to-patient ratio of 1:4. The Medical Telemetry Unit supports patients requiring continuous cardiac telemetry, with a nurse-to-patient ratio ranging from 1:5 to 1:6. Additionally, the Surgical Unit admits patients under the care of the internal medicine team for primary medical management with secondary surgical diagnoses. The Observation Unit manages patients admitted for short-term observation, typically 24 to 48 hours, with a nurse-to-patient ratio of 1:6.

The General Medicine Unit consists of 44 beds and is staffed by 60 nurses, ensuring a nurse-to-patient ratio of 1:4. The Intermediate Care Unit contains 20 beds and is supported by 41 nurses, maintaining a nurse-to-patient ratio of 1:3. When necessary, additional medicine patients are placed in overflow units throughout the hospital, which are not designated exclusively for medicine but provide supplementary capacity.

Interprofessional team and rounding protocol

The hospital medicine program consists of eight teams providing care for all hospitalized patients under the internal medicine service. The teams rotate on a seven-day cavitation cycle where they work for seven days and take off for seven days. Interdisciplinary rounds usually begin between 10 and 11 AM.

Each team meets on its unit and goes over the daily patient list prior to rounding. The hospital uses an automation platform (Qventus). Qventus provides the patient's name, age, gender, room number, primary diagnosis, anticipated day of discharge, clinical status (stable to discharge or not), length of stay (LOS), barriers to discharge, and things to do today. Nurses present their patients while physicians decide on the anticipated discharge date, clinical status, and clinical barriers. Case managers and social workers discuss social barriers and placement issues, if any. The clinical pharmacist monitors the rounding process and places orders after confirming with physicians. The nurse leader guides the process and summarizes the discussion. After discussing all assigned patients, physicians start bedside rounds with nurses and clinical pharmacists. Nurses provide a brief presentation at the bedside and all orders are reviewed again by physicians and pharmacists. Patients and their families are encouraged to actively engage in discussions and are afforded the opportunity to pose questions, fostering collaborative communication and shared decision-making.

Nurse leaders meet again with nursing staff and case managers at 2 PM to check the things-to-do list and follow up on what was discussed during rounds.

Participants

The study participants comprised all patients admitted under the internal medicine service, along with physicians, nursing staff, case managers, and clinical pharmacists. Participants were informed about the researcher’s credentials and the objectives of the study and were provided the option to voluntarily participate or withdraw at any stage.

Implementation time frame

All stakeholders (nursing, faculty, pharmacist, care managers, and nursing supervisors) had access to directly email the study investigator for any urgent questions or clarifications; otherwise, they were encouraged to discuss issues during a weekly meeting.

Data were collected for 30 days from July 5, 2022, to August 25, 2022. Teams met every Friday with the investigator to assess the study's progress and answer any questions.

Measurements

The primary outcomes were provider satisfaction, education, experience, and skill levels after performing IDRs. Primary outcomes were assessed by surveys. The survey's items were based on previous scholars’ qualitative work on the impact of interdisciplinary rounds on providers' satisfaction [1-7]. Surveys were distributed in person via paper copies and there was no follow-up with non-respondents to minimize selection bias. The survey is included in the Appendices.

Interventions

The study surveyed the impact of IDRs on providers' satisfaction, education, experience, and skill levels after performing IDRs.

Medical staff satisfaction with IDRs was measured through a survey on the impact of IDRs on their education, patient care, communications, outcomes, coordination, and teamwork. The survey's items were based on previous scholars’ qualitative work on the impact of interdisciplinary rounds on providers' satisfaction [1-7].

Medical staff were asked to complete an 11-question survey about the impact of IDRs on their educational experience. Physicians were asked to complete a survey on their last day of work (the last day of their 7 days on). The rest of the medical staff (nurses, pharmacists, case managers, nursing supervisors) were given surveys on Fridays since it is the last day of their workweek.

Data analysis 

Descriptive statistics were utilized to summarize the characteristics of the respondent groups. The nonparametric Wilcoxon rank-sum test was employed to compare mean values between groups. To evaluate the strength and direction of associations among variables across respondent groups, the Spearman rank correlation test was applied as a nonparametric measure of correlation. Nonparametric tests like the Wilcoxon rank-sum are chosen over parametric alternatives because the distribution of the data was unknown or suspected to not be normally distributed, meaning it was not possible to make strong assumptions about the population parameters, thus making a nonparametric approach more reliable in this case.

## Results

During the 30-day study period, a total of 100 surveys were distributed, with 72 surveys completed, yielding a 72% response rate. This included responses from 82% (37/45) of nursing staff, 71% (25/35) of attending physicians, 50% (5/10) of pharmacists, and 50% (5/10) of case managers. Table 1 presents the demographic characteristics of the survey respondents.

**Table 1:** Demographics of participants (N=72) N: Total number of respondents per group. Percentages indicate the proportion of the total sample.

Demographics of Participants (N=72)					
Healthcare provider	Nursing staff N=37 (51%)	Attending physicians N=25 (34%)	Pharmacist N=5 (7%)	Case managers N=5 (7%)	Full Sample N=72 (100%)
Gender					
Female	29	7	3	5	44
Male	8	18	2	0	28
Years in the role					
<1 year in their role	10	2	3	2	17
1-2 years in their role	3	13	1	0	17
>2 years in their role	24	10	1	3	38

The respondents’ perceptions of the benefits of IDRs are reported as mean values on a Likert scale ranging from 1 to 5, with 1 representing "Strongly Disagree" and 5 representing "Strongly Agree". These perceptions are summarized for the entire respondent pool as well as for each participant group (Table 2).

**Table 2:** Comparison of participant-reported ratings on the benefits of bedside interprofessional rounds Data Representation: The data are represented as mean values on a Likert scale from 1 to 5, with 1 = Strongly Disagree and 5 = Strongly Agree. The standard deviation (SD) is included to reflect the variability of responses. N: Total number of respondents per group. Percentages indicate the proportion of the total sample.

Comparisons of Ratings of the Benefits of Bedside Interprofessional Rounds as Reported by Participants							
Healthcare staff	Nursing N=37 (51%)	Attending Physicians N=25 (34%)	PharmacistN=5 (7%)	Case managers N=5 (7%)	Full sample N=72 (100%)	SD	Rank 1-13
Survey item	Mean Value						
Communication between nurses and physicians has improved after the implementation of Interdisciplinary rounds	5	5	4	5	4.75	0	1
Awareness of clinical issues needing to be addressed has improved after the implementation of Interdisciplinary rounds.	5	5	4	5	4.75	0	2
Team building between nurses and physicians has improved after implementing Interdisciplinary rounds.	5	5	4.6	4.2	4.7	0.56568542	3
Your medical education has improved after the implementation of Interdisciplinary rounds.	5	4.8	4.1	4	4.475	0.6363961	4
Coordination of the patient's care has improved after the implementation of Interdisciplinary rounds.	5	4.2	4	4.8	4.5	0.14142136	5
Nursing contribution to a patient's care plan has improved after the implementation of Interdisciplinary rounds.	5	4.2	4	4.8	4.5	0.14142136	6
Quality of care delivered in our unit has improved after the implementation of Interdisciplinary rounds.	4	4	5	5	4.5	0.70710678	7
Appreciation of the roles/contributions of other providers has improved after the implementation of Interdisciplinary rounds.	4	5	5	4	4.5	0	8
Shared decision-making between patients and providers has improved after implementing Interdisciplinary rounds.	3.5	4.9	4.8	4.5	4.425	0.70710678	9
Patients' satisfaction with their hospitalization has improved after the implementation of Interdisciplinary rounds.	3	4.9	4	4.5	4.1	1.06066017	10
Respect/dignity to patients has improved after the implementation of Interdisciplinary rounds.	5	5	4.5	5	4.875	0	11
Number of pages/phone calls between nurses and physicians has improved after the implementation of Interdisciplinary rounds.	4.5	4.5	5	4.1	4.525	0.28284271	12
Your clinical skills have improved after the implementation of Interdisciplinary rounds.	5	4.8	4.8	4	4.65	0.70710678	13

The highest-rated benefits pertained to communication, coordination, and teamwork, including:

“Improves communication between nurses and physicians”

“Improves awareness of clinical issues that need to be addressed”

“Enhances team-building between nurses and physicians”

In contrast, the lowest-rated benefits were associated with efficiency, process improvements, and clinical outcomes such as:

“Reduces patients’ length of hospital stay”

“Improves the timeliness of consultations”

“Minimizes the ordering of unnecessary tests and treatments”

**Figure 1:**
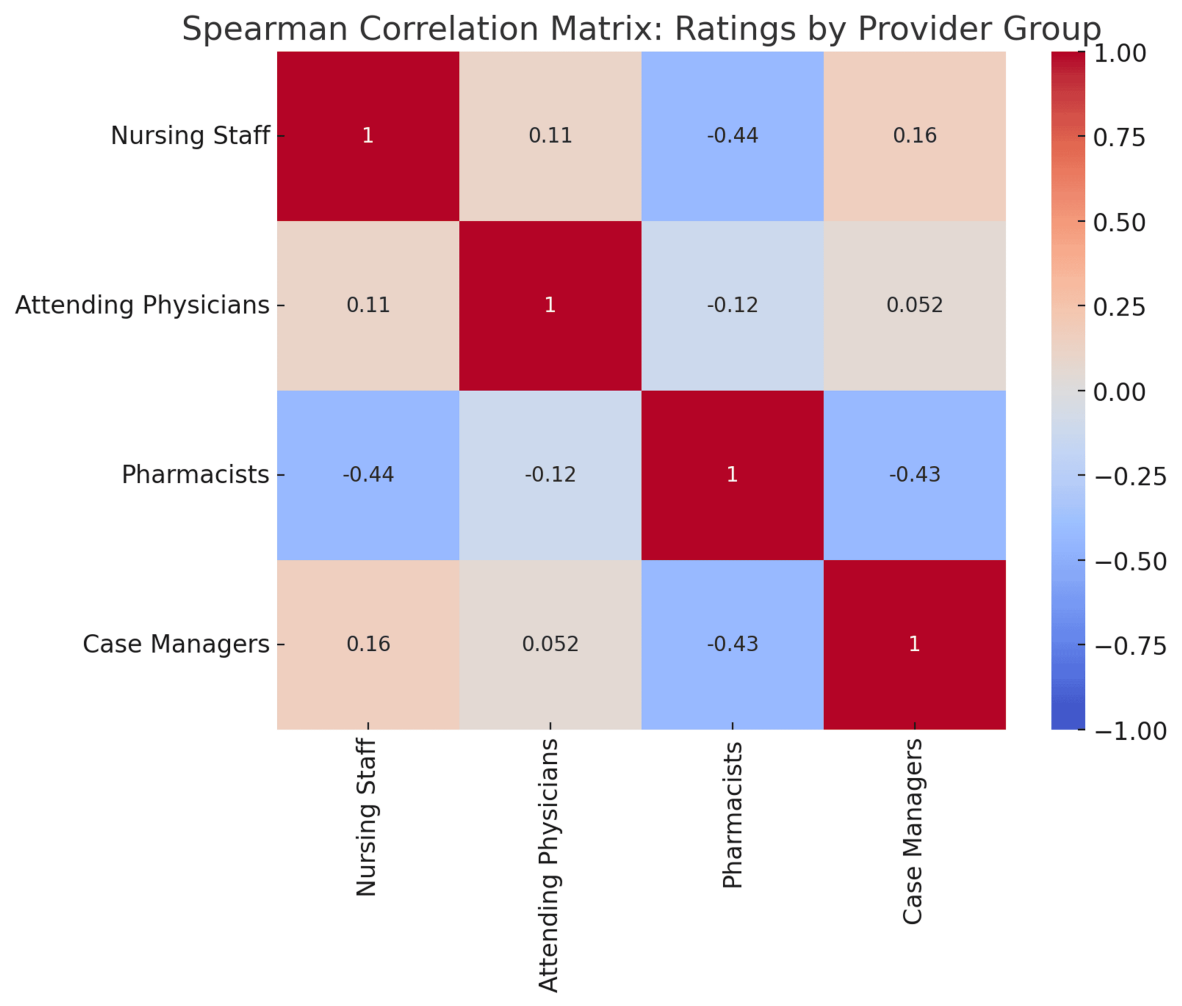
Spearman correlation matrix: ratings by Provider Group

The Spearman correlation matrix was generated to explore the relationships between the ratings provided by nursing staff, attending physicians, pharmacists, and case managers regarding the benefits of IDRs. The correlation coefficients are presented in Figure 1 and summarized below.

Key findings

A low positive correlation was observed between nursing staff and case managers (0.16), indicating some shared perceptions regarding the benefits of IDRs.

Similarly, nursing staff and attending physicians showed a weak positive correlation (0.11), suggesting some alignment in their ratings, likely related to clinical collaboration.

Pharmacists' ratings demonstrated a moderate negative correlation with both nursing staff (-0.44) and case managers (-0.43), highlighting divergent perceptions.

The correlation between attending physicians and case managers was minimal (0.05), indicating that these groups had little overlap in their views on the benefits of IDRs.

## Discussion

Our study aimed to compare the perceptions of nursing staff, physicians, case managers, and pharmacists on the benefits of IDRs. When we compared mean values among our participants' groups (nursing staff, attending physicians, pharmacists, and case managers), we found statistically significant differences between response rates for each item in the surveys. Our surveys showed that nursing staff had more favorable responses (agree: 4, strongly agree: 5) to all the items, except for patients’ satisfaction with their care and improvement of shared decision-making between patients and providers. We are not sure what were the exact reasons for that. However, this could be because of the nature of nursing staff work as nurses switch their patients' assignments more frequently than physicians. This could impact the nursing perspective on patients’ satisfaction with their care and the improvement of shared decision-making between patients and providers.

We also asked providers to rank the benefits of IDRs on a scale of 1 being the highest and 13 being the lowest. Highest-ranked benefits were related to communication, awareness of medical issues, team building, and medical education. Lowest-ranked benefits were related to patients' satisfaction with their care, improvement of patients’ respect/dignity, number of pages/phone calls between nurses and physicians, and improvement of clinical skills. Our results are consistent with previous studies that revealed that the highest benefits of IDRs are related to interprofessional communication and coordination [14-17].

The findings underscore notable similarities and differences in the perceptions of frontline healthcare providers. Both nurses and physicians reported a substantial positive impact of IDRs on their clinical skills, whereas case managers indicated a comparatively lower impact for the same item. This discrepancy may be attributed to the more direct, hands-on involvement of nurses and physicians in patient care. Physicians also reported the greatest impact of IDRs on their medical education, which could be linked to the high level of interaction and collaboration with other healthcare professionals, including nurses, pharmacists, and case managers. These results align with those of a previous study by Lopez et al., which found that resident physicians perceived structured IDRs as having a positive influence on their medical education [15].

The impact of IDRs on medical education was reported to be lowest by case managers, followed by pharmacists, with the highest ratings provided by nurses and physicians. Several factors may account for these differences. First, the role of case managers primarily focuses on patient disposition, addressing social aspects of care, and managing post-discharge challenges. While these responsibilities are critical to patient care, they do not typically involve direct engagement with the medical aspects of treatment. Second, pharmacists play a crucial role in medication management, including medication reconciliation at admission and discharge, identifying potential drug interactions, and adjusting complex medication regimens. However, their contributions, though essential, do not directly impact the diagnostic or prognostic components of patient care. In contrast, the educational framework of nurses emphasizes a patient-centered approach, which may align more closely with the objectives of IDRs.

This study has several limitations. The investigation was conducted at a single community hospital and the short-term nature of the data collection (only 30 days), limiting the generalizability of the findings. The varying levels of response rates across participant groups (e.g., 82% for nurses vs. 50% for case managers) may have influenced the outcomes or introduced response bias. Furthermore, as IDRs are a strategic priority within the institution, perceptions of their benefits and barriers may differ in hospitals where similar initiatives are not actively promoted. Although the survey used in this study underwent pilot testing to ensure content relevance, it was not formally validated. Additionally, despite the anonymity of responses, the potential for social desirability bias remains, which may have affected the accuracy of the results.

The correlation analysis reveals several important insights into how different healthcare provider groups perceive the benefits of IDRs. The low positive correlations between nursing staff, attending physicians, and case managers suggest that, although these groups recognize some shared benefits, their experiences and priorities within the IDR framework vary.

The negative correlations involving pharmacists highlight the distinct nature of their role, which focuses primarily on medication management rather than direct patient care coordination or communication. This divergence indicates that pharmacists may not experience the same level of educational or teamwork benefits from IDRs as other provider groups.

The minimal correlation between attending physicians and case managers reflects the differing nature of their responsibilities. While physicians focus primarily on clinical care and education, case managers are more involved in discharge planning and the social aspects of patient care, which may explain their lower alignment in rating the benefits of IDRs.

## Conclusions

Our study offers valuable insights into healthcare providers' perceptions of Interdisciplinary Bedside Rounds (IDRs). The findings provide actionable information to guide the structure, content, and delivery of IDRs, enhancing their effectiveness and benefiting both patients and healthcare providers. Participants highlighted several key advantages of IDRs, including improvements in medical education, team-building, and communication. However, the study also identified areas requiring attention such as tailoring IDRs to better accommodate the perspectives of all professional groups and addressing barriers to participation for pharmacists and case managers. To strengthen the implications of our findings, it is important to address potential selection and response biases, particularly given the variable response rates among different professional groups. Future research should include strategies to mitigate these biases, such as ensuring more inclusive sampling and exploring non-respondents’ perspectives. Additionally, the generalizability of our results is limited by the study’s context - a single community hospital. Comparing our findings with data from larger academic institutions or rural facilities could offer a broader understanding of how IDRs function in diverse healthcare settings.

A more critical analysis of the negative or neutral findings, such as lower ratings for efficiency-related benefits, suggests that IDRs may need modifications to achieve their intended outcomes. These findings can guide future research or implementation strategies by highlighting specific aspects of IDRs that require refinement. For example, addressing efficiency concerns through streamlined workflows or targeted training could enhance the perceived value of IDRs across all provider groups. Future research should also explore the long-term impact of IDRs on medical education, the development of clinical skills, and the overall experience of healthcare providers. These investigations will be essential to fully understand the enduring effects of IDRs on both provider performance and patient care, ensuring their continued relevance and optimization in varying healthcare environments.

## Appendices

The questionnaire: The impact of patient-centered structured interdisciplinary bedside rounds on medical staff satisfaction, education, and experience

Your role:

o   o Pharmacist 

o   Nursing staff

o   Case worker 

o   Physician 

o   Other: 

How long have you been in this role?

o   <1 year

o   1-2 years  

o   2 years 

Please choose one answer for the following items. How do you think Interdisciplinary rounds are affecting the following?

Communication between nurses and physicians. 

o   Strongly disagree 

o   Disagree 

o   Neither agree nor disagree 

o   Agree 

o   Strongly agree

Awareness of clinical issues needing to be addressed.

o   Strongly disagree 

o   Disagree 

o   Neither agree nor disagree 

o   Agree 

o   Strongly agree

Team-building between nurses and physicians.

o   Strongly disagree 

o   Disagree 

o   Neither agree nor disagree 

o   Agree 

o   Strongly agree

Coordination of the patient's care.

o   Strongly disagree 

o   Disagree 

o   Neither agree nor disagree 

o   Agree 

o   Strongly agree

Nursing contributions to a patient's care plan.

o   Strongly disagree 

o   Disagree 

o   Neither agree nor disagree 

o   Agree 

o   Strongly agree

Quality of care delivered in our unit.

o   Strongly disagree 

o   Disagree 

o   Neither agree nor disagree 

o   Agree 

o   Strongly agree

Appreciation of the roles/contributions of other providers.

o   Strongly disagree 

o   Disagree 

o   Neither agree nor disagree 

o   Agree 

o   Strongly agree

Shared decision-making between patients and providers.

o   Strongly disagree 

o   Disagree 

o   Neither agree nor disagree 

o   Agree 

o   Strongly agree

Patients' satisfaction with their hospitalization.

o   Strongly disagree 

o   Disagree 

o   Neither agree nor disagree 

o   Agree 

o   Strongly agree

Respect/dignity to patients.

o   Strongly disagree 

o   Disagree 

o   Neither agree nor disagree 

o   Agree 

o   Strongly agree

Number of pages/phone calls between nurses and physicians. 

o   Strongly disagree 

o   Disagree 

o   Neither agree nor disagree 

o   Agree 

o   Strongly agree

Educational opportunities for house staff/students.

o   Strongly disagree 

o   Disagree 

o   Neither agree nor disagree 

o   Agree 

o   Strongly agree

Efficiency of your work.

o   Strongly disagree 

o   Disagree 

o   Neither agree nor disagree 

o   Agree 

o   Strongly agree

Adherence to evidence-based guidelines or interventions.

o   Strongly disagree 

o   Disagree 

o   Neither agree nor disagree 

o   Agree 

o   Strongly agree

The accuracy of your sign-outs (or reports) to the next shift.

o   Strongly disagree 

o   Disagree 

o   Neither agree nor disagree 

o   Agree 

o   Strongly agree

Ordering of unnecessary tests and treatments.

o   Strongly disagree 

o   Disagree 

o   Neither agree nor disagree 

o   Agree 

o   Strongly agree

Patients' hospital length of stay.

o   Strongly disagree 

o   Disagree 

o   Neither agree nor disagree 

o   Agree

o   Strongly agree
